# Pilot Study of Grape Pomace Extract Impact on *Nosema ceranae*-Infected Honey Bees

**DOI:** 10.3390/insects17080864

**Published:** 2026-08-19

**Authors:** Uroš Glavinić, Stefan Jelisić, Marko Ristanić, Đura Nakarada, Branislav Vejnović, Milivoje Ćosić, Zoran Stanimirović

**Affiliations:** 1 Faculty of Veterinary Medicine, Department of Biology, University of Belgrade, 11000 Belgrade, Serbia; 2 Faculty of Physical Chemistry, Center for Physical Chemistry of Biological Systems, BioScope Labs, University of Belgrade, 11158 Belgrade, Serbia; 3 Faculty of Veterinary Medicine, Department of Economics and Statistics, University of Belgrade, 11000 Belgrade, Serbia; 4 Institute of Forestry, 11030 Belgrade, Serbia

**Keywords:** honey bee, *Nosema ceranae*, grape pomace extract, oxidative stress, immune response

## Abstract

*Nosema ceranae* infection represents one of the major health threats to honey bees (*Apis mellifera*), contributing to colony weakening, oxidative stress, immune suppression, and increased mortality. Due to restrictions on the use of fumagillin and the growing demand for natural alternatives, this pilot study evaluated the potential of grape pomace (GP) extract (at a concentration of 200 μg/g) as a dietary supplement for improving honey bee health and controlling *Nosema ceranae* infection. Newly emerged worker bees were experimentally infected with *N. ceranae* and maintained under laboratory cage conditions for 15 days. Bees were supplemented with GP extract either preventively, simultaneously with infection, or after infection. Survival, spore loads, and expression of immune-, antioxidant-, and detoxification-related genes were evaluated. Results showed that *N. ceranae* infection significantly reduced bee survival and induced strong oxidative and immune stress responses. Supplementation with GP extract significantly improved survival and reduced spore loads in infected bees, particularly when administered preventively or at the onset of infection. The extract also modulated the expression of antioxidant-related genes, including CAT, Cu/ZnSOD, GST, and MnSOD, indicating attenuation of infection-associated oxidative stress. In non-infected bees, GP supplementation stimulated the expression of immune-related genes, especially *defensin* and *hymenoptaecin*, while such immunostimulatory effects were not fully maintained in infected groups. The findings suggest that grape pomace extract acts primarily through antioxidant and gut health-associated mechanisms rather than direct immune protection during infection. Overall, grape pomace extract demonstrated promising potential as a natural dietary supplement for improving honey bee resilience and reducing the impact of *Nosema ceranae* infection. Further studies with more concentrations and temporal dynamics need to be evaluated.

## Simple Summary

Honey bees are essential pollinators, but their health is increasingly threatened by diseases and environmental stressors. One of the most important honey bee pathogens is *Nosema ceranae*, a microscopic parasite that weakens bees, reduces their lifespan, and contributes to colony losses. Because the use of conventional treatments such as fumagillin is restricted in many countries, there is a growing need for safe and natural alternatives. In this study, we evaluated whether an extract obtained from grape pomace, a by-product of wine production, could improve honey bee health and help control *Nosema ceranae* infection. Honey bees were fed the extract before, during, or after experimental inoculation. Bees receiving the extract showed improved survival and lower levels of infection compared with untreated infected bees. The extract also reduced indicators of oxidative stress, suggesting that it helped bees better cope with the physiological damage caused by the parasite. The strongest benefits were observed when the extract was provided before or at the time of inoculation. These findings indicate that grape pomace extract may represent a sustainable and environmentally friendly dietary supplement for supporting honey bee health, which needs to be further evaluated.

## 1. Introduction

Honey bee colonies (*Apis mellifera* L.) play a fundamental role in both agricultural production and natural ecosystems due to their contribution to pollination and the provision of nutrient-rich food sources [1]. Nevertheless, in recent decades, significant colony losses have been recorded globally, creating serious economic and ecological concerns. Numerous stressors contribute to colony weakening, including pathogens and parasites, environmental contaminants, nutritional imbalances, and inadequate beekeeping management practices [2]. Among the most prevalent diseases of adult honey bees is nosemosis, caused by microsporidia of the genus *Nosema* [3]. In *A. mellifera*, infections with *N. apis* and *N. ceranae* species are identified, while *N. ceranae* is recognized as the predominant agent responsible for chronic infections and colony decline [4,5]. The reclassification of *Nosema apis* and *Nosema ceranae* into *Vairimorpha* proposed by Tokarev et al. [6] has been disputed by Bartolomé et al. [7] and most recently supported, with a compromise on disease naming, by Bojko et al. [8]; as regulatory bodies (WOAH, EFSA, USDA) and many leading researchers and journals in the field of apiculture continue to use *Nosema*, this name is retained throughout this study. This microsporidium primarily targets the epithelial cells of the midgut, and its DNA or presence has also been reported in other tissues and in hemolymph [9,10,11]. Damage to the digestive epithelium disrupts metabolic processes, contributing to malnutrition, diarrhea, reduced honey production, and increased winter mortality [12]. Moreover, *N. ceranae* infection can alter carbohydrate metabolism, induce oxidative stress, compromise immunity, and exacerbate the harmful effects of pesticides [3].

The only registered product historically used for nosemosis control is fumagillin, an antibiotic derived from *Aspergillus fumigatus*. However, its application has been banned in most European countries due to concerns related to residue accumulation in honey and other bee products, food safety risks, and negative effects on bee health. Despite remaining licensed in some countries such as the United States, Canada, and Argentina, its efficacy has been questioned [13]. Parallel to the general trend of reducing antibiotic use in beekeeping, the need for alternative treatments has driven extensive research into natural, safe, and residue-free substances [13,14].

Various natural compounds, including plant extracts, probiotics, polysaccharides, amino acid or vitamin supplements, and organic substances, have shown potential in reducing *Nosema* spore loads or enhancing the overall physiological condition of bees [12,13,15,16,17,18,19,20,21,22]. Of particular interest are bioactive molecules derived from plants, which are known for their nutritive and medicinal properties. Among the various extracts derived from plants and their anatomical components, those obtained from grape pomace—GP (the residual biomass generated after pressing grapes for winemaking) are of particular scientific and industrial interest. This relevance results not only from the well-documented bioactive potential of grape pomace extracts [23,24,25], but also from the broader sustainability implications associated with valorizing this oenological by-product [26]. The systematic utilization of grape pomace contributes to waste minimization within the wine industry and aligns with contemporary principles of environmental protection and circular bioeconomy strategies [27].

Recent research has indicated that extracts derived from grape pomace may hold significant potential for applications in apicultural health management. In vitro studies have demonstrated that hydroethanolic extracts of grape pomace contain diverse flavonoids, anthocyanins, and stilbenes, which exhibit strong antioxidant activity and the capability to modulate cellular redox status in honey bee cell cultures (AmE-711 cell line), protecting against oxidative stress at the cellular level [28]. Furthermore, dietary supplementation of honey bees with grape pomace powder has been associated with a reduction in deformed wing virus load and an enhanced expression of immune-related genes, suggesting immunomodulatory benefits in vivo [29]. Collectively, these findings support the hypothesis that grape pomace extracts could serve as functional dietary supplements in beekeeping, contributing to honey bee colony resilience and overall health.

Motivated by these promising findings, the present study was designed to conduct controlled laboratory (cage) experiments aimed at establishing optimal application protocols for a grape pomace-based extract in honey bees (*Apis mellifera*). Specifically, we sought to evaluate the supplement’s potential to stimulate immune responses and to assess its efficacy in mitigating infection by *Nosema ceranae* under standardized experimental conditions.

## 2. Materials and Methods

### 2.1. Experimental Design

A controlled laboratory experiment was conducted to evaluate the effects of a grape pomace-based extract on honey bees (*Apis mellifera*) experimentally infected with *Nosema ceranae* and kept for 15 days in cages. Newly emerged worker bees were obtained from brood frames collected from clinically healthy colonies (regularly checked by an experienced veterinary specialist, one of the coauthors of this work), maintained at the institutional apiary (Department of Biology, Faculty of Veterinary Medicine, University of Belgrade). Frames containing sealed brood were incubated at 34 ± 1 °C and 60 ± 5% relative humidity. Brood were collected from multiple healthy colonies, and newly emerged bees were subsequently pooled before being randomly allocated to the experimental cages [30]. Each group was established with three replicates (cages).

Bees were maintained in specially designed cages [30], each containing 80 individuals. Cages were kept in an incubator under controlled conditions (34 ± 1 °C; 60 ± 5% RH; darkness). Bees were supplied *ad libitum* with 50% (*w*/*v*) sucrose solution (supplemented with extract according to experimental design—see chapter *Experimental Infection and Treatment Protocol*) throughout the experiment.

### 2.2. Extract Preparation

Grape pomace from the Prokupac variety (*Vitis vinifera* L.) was sourced from a winery in Vlaški Do, Serbia, following the completion of the winemaking process. The pomace material, comprising skins, seeds, and stems, was freshly collected and transported to the laboratory under cold-chain conditions. The fresh pomace was freeze-dried (lyophilized) for 48 hh using a laboratory-scale lyophilizer (Biobase, Jinan, China). The resulting lyophilized material was milled into a homogeneous powder with an electric grinder (Siemens, Munich, Germany) and subsequently stored at −80 °C until extraction.

Bioactive compound extraction was performed via pressurized liquid extraction (PLE). A quantity of 50 g of powdered pomace was packed into a cotton bag and loaded into the extraction vessel of a Superex F500 system (Superex, Karatay/Konya, Turkey). Aqueous ethanol (50%, *v*/*v*) was used as the extraction solvent [31], applied at a solvent-to-solid ratio of 5:1 (mL/g). The extraction was performed at 200 bar and 40 °C, with the process comprising a static phase of 30 min followed by a dynamic phase of 60 min. Once the first cycle was complete, the extract was collected and a second extraction cycle was carried out under identical conditions using fresh solvent, in order to maximize the recovery of bioactive constituents. The pooled extracts were then freeze-dried to yield a dry powder representing 6.4% of the original dry pomace mass. The resulting extract was kept at −80 °C until further analyses.

### 2.3. Preparation of Nosema ceranae Inoculum

*Nosema ceranae* spores were isolated from naturally infected adult bees. The presence of *N. ceranae* and the absence of other species were confirmed by light microscopy and molecular diagnostics [12,13]. Midguts were dissected, homogenized in distilled water, and filtered through sterile gauze to remove debris. The suspension was centrifuged, and the pellet was resuspended in water. Spore concentration was determined using a hemocytometer under microscopy and adjusted to the inoculation dose of 10^6^ spores/mL.

### 2.4. Experimental Infection and Treatment Protocol

On day 3 post-emergence, bees assigned to infected groups were inoculated with sucrose solution containing *N. ceranae* spores. Control bees received the same volume of pure sucrose solution without spores.

**Scheme 1:**
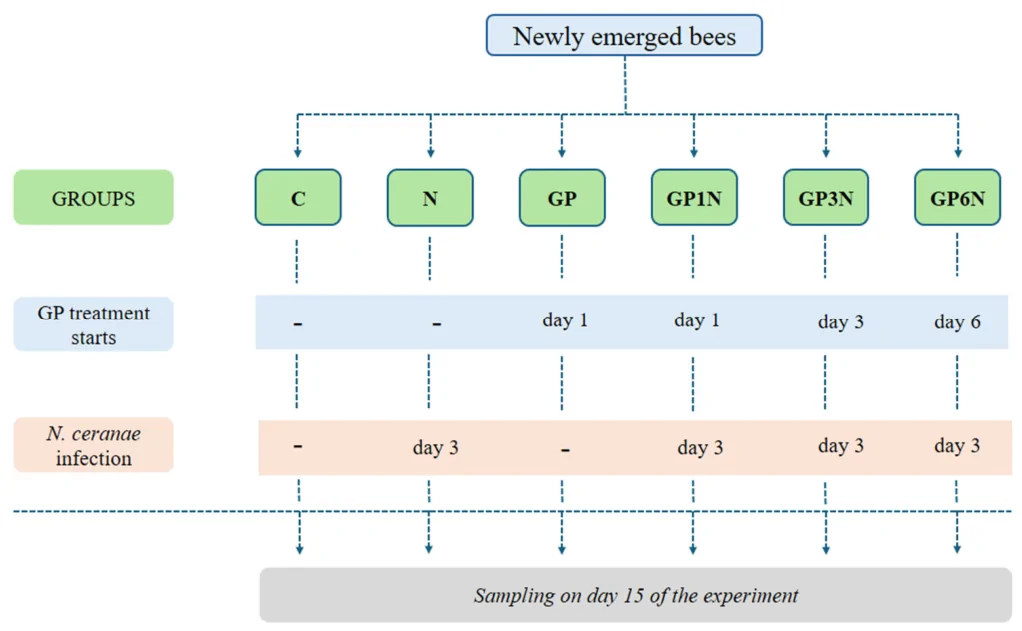
Experimental design. C, non-infected, non-treated control group; N, *N. ceranae*-infected and untreated group; GP, non-infected group treated with grape pomace extract; GP1N, GP3N, and GP6N, infected groups treated with grape pomace extract starting on day 1, day 3, and day 6 post-emergence, respectively.

The test extract was administered via sucrose solution at a single concentration of 200 μg/g (0.2 mg/g) based on our previous in vitro findings [28,31]. Fresh feeding solutions were prepared daily. Except for the non-infected and non-treated control group (C) and *N. ceranae*-infected control group (N), the experiment included the following treatment groups (Scheme 1): a group treated with extract but not infected with *Nosema* (GP); and those infected with *Nosema* spores and treated with extract from day 1 (GP1N), day 3 (GP3N), and day 6 (GP6N).

### 2.5. Survival Monitoring

Bee mortality was recorded daily throughout the experimental period. Dead individuals were removed from cages, and cumulative mortality was calculated for each group. Survival data were used for subsequent statistical analysis.

### 2.6. Assessment of Nosema Infection Intensity

On day 15 of the experiment, 10 bees from each cage were collected for assessment of *Nosema* infection intensity according to the previously described procedure [30]. Briefly, abdomens were homogenized individually in 1 mL of water. Spore loads were quantified using a hemocytometer and expressed as the number of spores per bee [32].

### 2.7. Gene Expression Analyses

Honey bee samples obtained from cages on day 15 of the experiment were processed by maceration of individual bees in Qiagen TissueLyser II (Qiagen Inc., Hilden, Germany). From each bee, total RNA was extracted using the Quick-RNA MiniPrep Kit (Zymo Research, Irvine, CA, USA) according to the manufacturer’s instructions. RNA quantity and quality were assessed spectrophotometrically, and only RNA samples meeting the predefined quality criteria (A260/A280 ratios between 1.9 and 2.1 and satisfactory RNA concentrations) were included in the study.

The expression of the genes (Table 1) was quantified by real-time PCR (qPCR) using complementary DNA (cDNA) synthesized from the extracted RNA using FastGene Scriptase (Nippon Genetics Europe, Düren, Germany) according to the manufacturer’s instructions and using random hexamer primers. Amplification was performed in a 36-well rotor using Rotor-Gene Q 5plex (Qiagen Inc., Hilden, Germany) with FastGene^®^ IC Green 2 qPCR Universal Mix (Nippon Genetics Europe, Düren, Germany). The thermal cycling protocol was performed according to the manufacturer’s instructions and optimized based on our previous studies [33]. Primer sequences used for qPCR are listed in Table 1. Gene expression levels were normalized against the reference gene β-actin. The qPCR experiments were designed, optimized, performed, and reported in accordance with the Minimum Information for Publication of Quantitative Real-Time PCR Experiments (MIQE) guidelines. Primer specificity was confirmed by the presence of a single peak in the melting curve analysis and by the absence of amplification in no-template controls (NTCs). Standard curves generated from serial cDNA dilutions were used to determine amplification efficiency and linearity for each primer pair. Amplification efficiencies ranged from 95.1% to 103.8%, with correlation coefficients (R^2^) exceeding 0.99 for all assays. Technical replicates showed high reproducibility. The stability of the reference gene (β-actin) was verified across all experimental groups prior to normalization. Relative gene expression was calculated using the 2^−ΔΔCt^ method, as amplification efficiencies of the target and reference genes were comparable, while the median value of the gene expression level in the C group served as a calibrator and was calculated using the 2^−ΔΔCt^ method [33] (Glavinic et al., 2022). Each biological sample was analyzed in technical replicates.

### 2.8. Statistical Analysis

The assessment of bee survival was conducted by monitoring the daily count of dead bees within each experimental group. The data on survival distribution were obtained via the Kaplan–Meier survival estimator and compared using the log-rank test. The results of *N. ceranae* spore loads and expression levels of the genes Abaecin, Defensin, Hymenoptaecin, Vitellogenin, CAT, CuZnSOD, GST, and MnSOD were tested for normality using the Shapiro–Wilk’s test. As the data for *N. ceranae* spore loads and gene expression levels were not normally distributed (Shapiro–Wilk’s test, *p* < 0.05), the results were compared using the Kruskal–Wallis test followed by Dunn’s post hoc test. Significant differences were estimated at *p* < 0.05, *p* < 0.01, *p* < 0.001, and *p* < 0.0001 significance levels. Statistical analysis was carried out using statistical software GraphPad Prism version 7 (GraphPad, San Diego, CA, USA).

## 3. Results and Discussion

**Figure 1:**
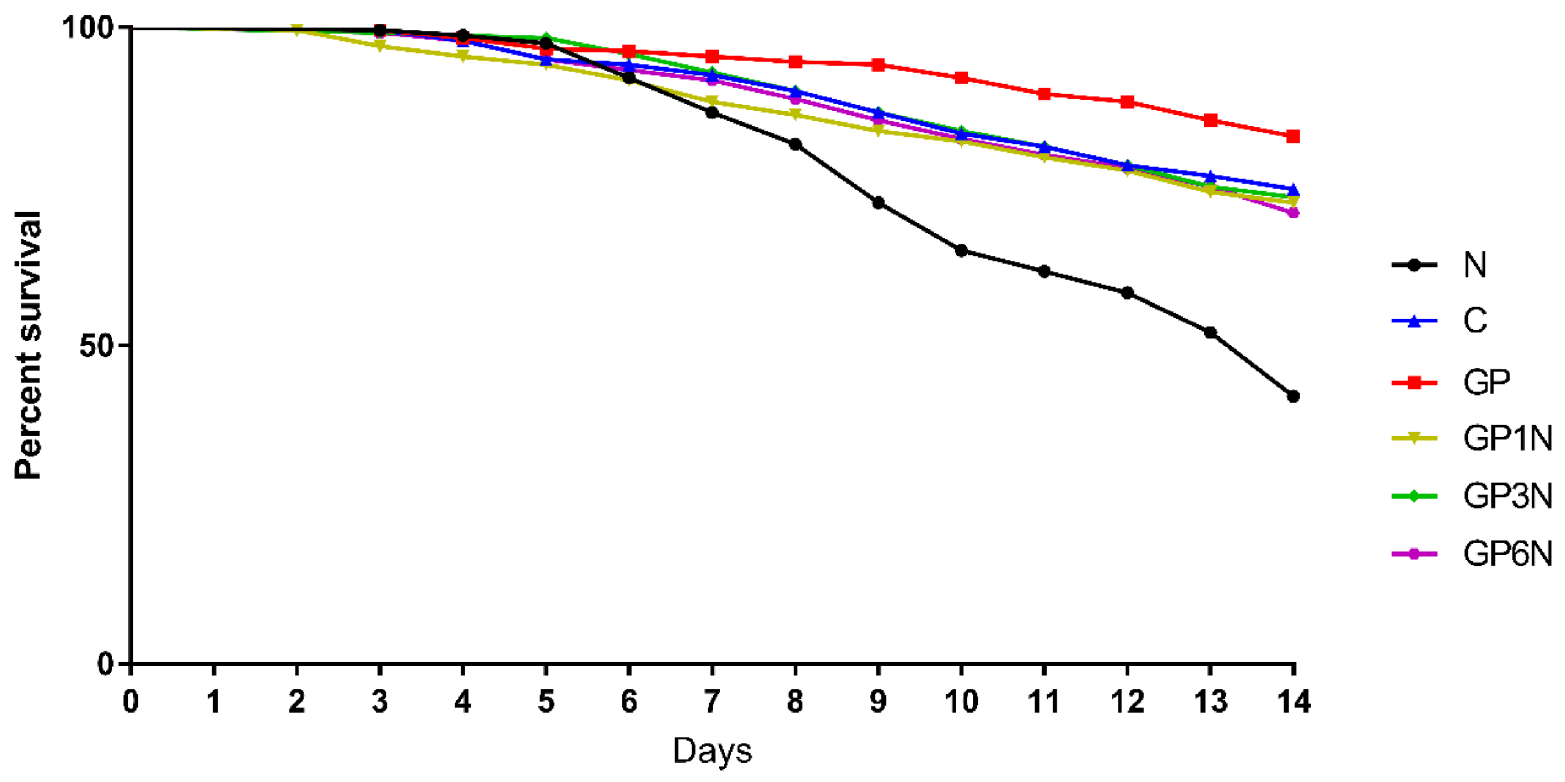
Survival curves of bees from control groups and treated groups. Control (non-infected) group (C), group infected with *N. ceranae* (N), non-infected but treated with grape pomace (GP), groups infected with *N. ceranae* and treated from day 1 (GP1N), day 3 (GP3N), and day 6 (GP6N). Survival distributions were estimated using the Kaplan–Meier method and compared among groups using the log-rank test.

*Nosema ceranae* infection in our experiment induced significant mortality. Mortality was obvious in the N group (*Nosema*-infected control) being statistically significant (*p* < 0.001) compared to all other experimental groups (Figure 1). This confirms a negative effect of *N. ceranae* on bee survival, which was proven in previous similar studies [12,13,21,30,33,37,38,39]. On the other hand, supplementation with GP extract improved bee survival, with the GP group showing significantly higher survival rates (*p* < 0.05) compared to all other groups, including the control (C) group (Figure 1). These effects may be attributed to different mechanisms, which should be analyzed in correlation with other parameters monitored in this study, as discussed below.

**Figure 2:**
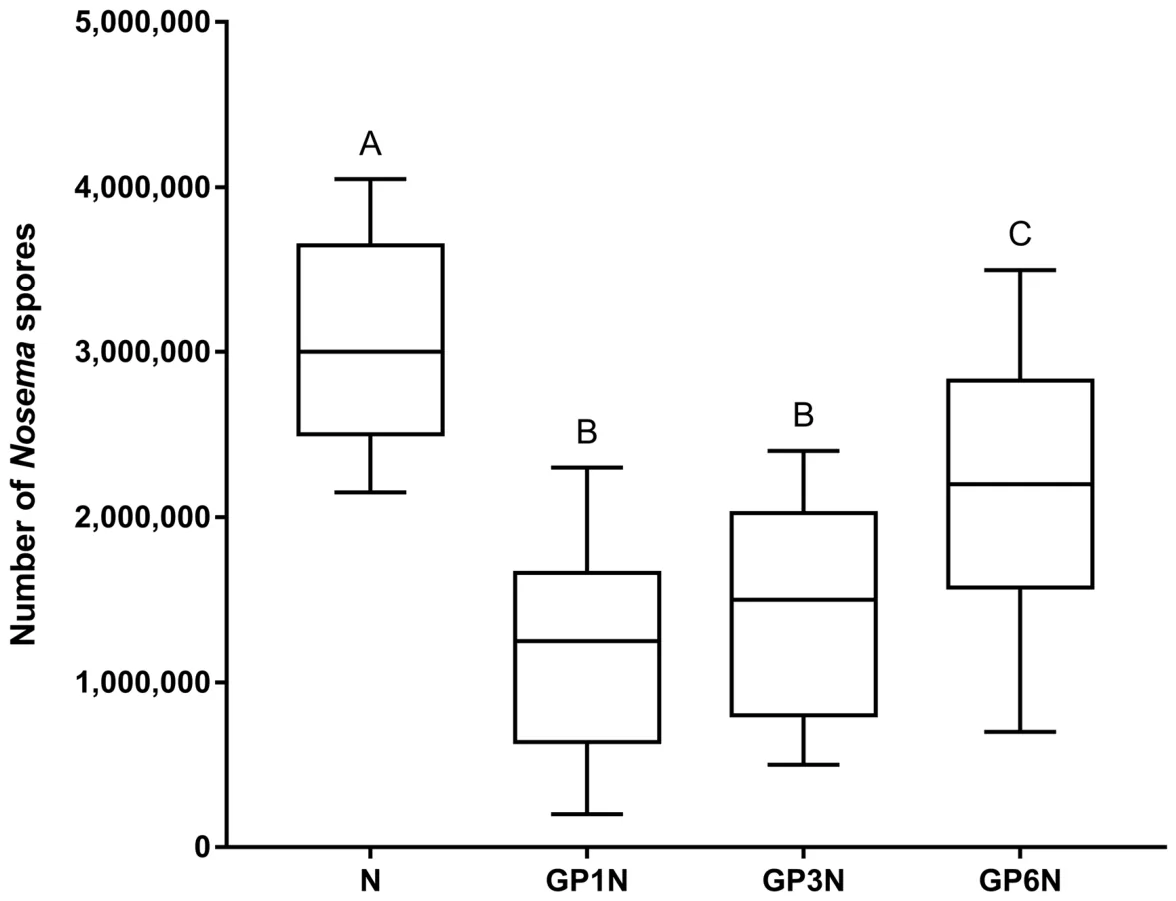
*N. ceranae* spore loads in bees from the group infected with *N. ceranae* (N) and groups infected with *N. ceranae* and treated from day 1 (GP1N), day 3 (GP3N), and day 6 (GP6N). Statistical differences among groups were assessed using the Kruskal–Wallis test followed by Dunn’s multiple comparison test. Different uppercase letters above box plots indicate significant differences among groups (*p* < 0.05).

*Nosema* spores (Figure 2) were detected in all experimentally infected groups (N, GP1N, GP3N, and GP6N), while in non-infected control groups (C and GP) bees remained non-infected until the end of the experiment, confirming that the newly emerged bees were *Nosema*-negative and that no cross-contamination occurred during the experiment. The number of *Nosema* spores in the *Nosema*-infected control group was significantly higher (*p* < 0.01) compared to all other *Nosema*-positive groups (GP1N, GP3N, and GP6N). This finding reveals the potential of the tested extract in reducing *Nosema* infection. Even though the exact mechanism of the potential antimicrosporidian effect is currently not known, numerous previous studies have demonstrated GP extract’s antimicrobial effect [25,40,41,42,43,44,45]. The antimicrobial effect was also demonstrated in the gut and correlated with the promotion of probiotic bacterial growth [43], which could represent a possible mechanism underlying honey bee resistance against *Nosema* infection.

Analysis of spore counts (Figure 2) in the group infected and supplemented with extract showed that the highest number was in group GP6N, being significantly (*p* < 0.05) higher than in the other two groups (GP1N and GP3N), but still lower (*p* < 0.01) compared to the *Nosema* control (N) group. This shows that GP extract has a better effect when applied preventively (GP1N) or at the same time as infection (GP3N) than as a post-inoculation treatment (GP6N). This confirms that GP extract has preventive effects, functioning as probiotic and prebiotic components [40,43], inhibiting the growth of opportunistic microorganisms [41], reestablishing the intestinal barrier function and inducing production of beneficial metabolites [46], or improving gut health by other mechanisms [45].

**Figure 3:**
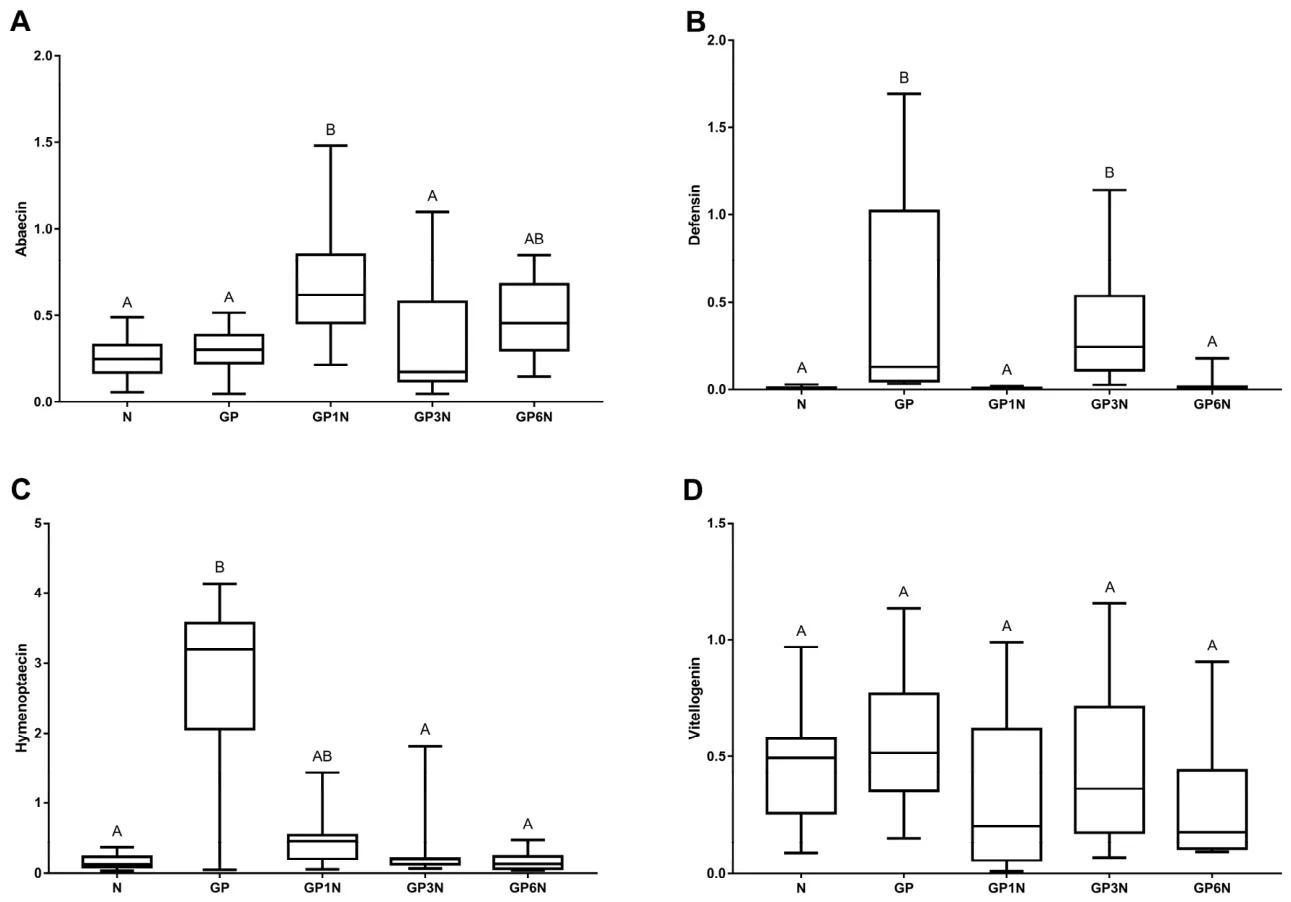
Expression levels for immune-related genes: (**A**) Abaecin, (**B**) Defensin, (**C**) Hymenoptaecin, and (**D**) Vitellogenin. Group treated with grape pomace (GP), *N. ceranae*-infected control (N), and groups infected and treated with GP from day 1 (GP1N), day 3 (GP3N), and day 6 (GP6N). Group names are presented in Table 1. Statistical differences among groups were assessed separately for each gene using the Kruskal–Wallis test followed by Dunn’s multiple comparison test. Different uppercase letters above box plots indicate significant differences among groups within each panel (*p* < 0.05).

When analyzing the expression levels of the immune-related genes in experimental groups (Figure 3), the highest expression of hymenoptaecin and defensin genes was detected in the GP group, which received grape pomace (without *Nosema* infection), being significantly higher (*p* < 0.01) than in all other groups (N, GP1N, GP3N, and GP6N). The stimulative effect of a supplement made from *Agaricus blazei* mushroom was recorded for these two genes in the study of Glavinic et al., ref. [13] in which, besides immune stimulation, supplementation induced a decline in *Nosema* infection. The present findings indicate that *Nosema* induces suppression of immune-related genes, which is consistent with the previous studies [12,13,21,30,33,47,48,49]. However, the applied extract did not prevent this suppression when applied to infected bees (groups GP1N, GP3N, and GP6N), suggesting that its mechanism of action is more immunostimulatory than immunoprotective as a therapeutic support for infected bees, which has been observed for some other supplements as well [3,21]. Contrary to other genes, expression of genes for vitellogenin was not significantly different (*p* > 0.05) among the experimental groups (Figure 3D). Vitellogenin is a lipoprotein that ensures the development of the fat body in bees [50]; its synthesis depends on the availability and quality of food, and it has a direct effect on the foraging behavior of bees [51]. Our findings could be explained by the nature of the laboratory (cage) experiment in which some social activities such as nursing, foraging, etc. could not be performed [52], as well as the specific character of grape pomace as a food additive. Other diets were tested as a protein source and also did not have an impact on vitellogenin content in the hemolymph [53].

**Figure 4:**
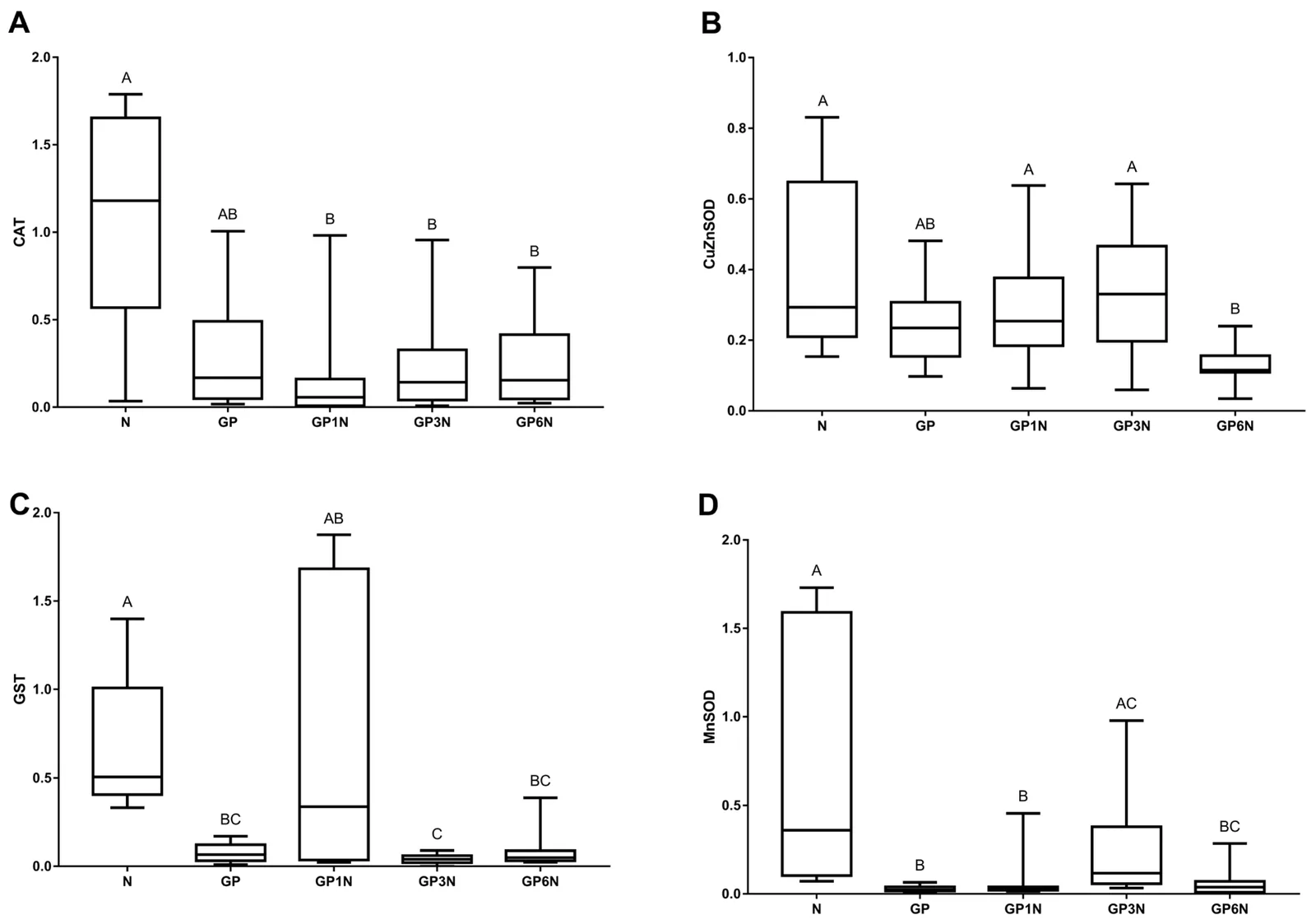
Expression levels for antioxidant and detoxification-related genes: (**A**) catalase (CAT), (**B**) copper/zinc superoxide dismutase (Cu/Zn SOD), (**C**) glutathione S-transferase (GST), and (**D**) mitochondrial manganese superoxide dismutase (MnSOD). Group treated with grape pomace (GP), *N. ceranae*-infected control (N), and groups infected and treated with GP from day 1 (GP1N), day 3 (GP3N), and day 6 (GP6N). Group names are presented in Table 1. Statistical differences among groups were assessed separately for each antioxidant enzyme using the Kruskal–Wallis test followed by Dunn’s multiple comparison test. Different uppercase letters above box plots indicate significant differences among groups within each panel (*p* < 0.05).

The expression profile of genes for catalase (CAT) revealed a marked response of honey bees to *Nosema ceranae* infection. The infected control group (N) exhibited the highest CAT expression among the experimental groups (Figure 4A), indicating a pronounced activation of catalase-related antioxidant defense in bees exposed to infection without supplementation. Since catalase is involved in the decomposition of hydrogen peroxide, its increased expression in the N group suggests that *N. ceranae* infection imposed a considerable oxidative burden on infected bees [54,55]. In contrast, all grape pomace-supplemented and *Nosema*-infected groups showed lower CAT expression compared with the infected control (Figure 4A). This reduction was most evident in groups that received the extract before or at the time of infection (GP1N and GP3N), suggesting that grape pomace supplementation may attenuate the infection-induced need for strong catalase upregulation. These findings are in accordance with Karastergiou et al., ref. [24] who stated that CAT activities help prevent oxidative damage in cells, reduce the risk of chronic diseases, and promote overall health. The non-infected GP group in our experiment also maintained low CAT expression, indicating that the extract itself did not induce a pronounced oxidative stress response in healthy bees, which was proven for wormwood and oak bark-based supplement [21]. Taken together, the CAT expression pattern suggests that *N. ceranae* infection strongly activates peroxide-related antioxidant defense, whereas grape pomace extract appears to modulate this response and reduce the oxidative stress associated with infection, similarly to the previous studies [21,56].

The expression levels of the gene for Cu/ZnSOD (Figure 4B), encoding cytosolic copper/zinc superoxide dismutase, showed a complex response across experimental groups. The infected control group (N) displayed elevated and relatively variable Cu/ZnSOD expression, consistent with the activation of cytosolic antioxidant defense mechanisms in response to *N. ceranae*-induced oxidative stress. This response is biologically expected, as superoxide dismutase represents the first enzymatic line of defense against superoxide radicals [56,57]. Among the grape pomace-treated groups, Cu/ZnSOD gene expression levels varied depending on the timing of supplementation. The GP3N group showed relatively preserved expression compared with other treated groups, which may indicate that supplementation initiated at the time of infection maintained a controlled but functional cytosolic antioxidant response [28]. In contrast, the GP6N group showed the lowest Cu/ZnSOD gene expression (Figure 4B), suggesting that delayed supplementation may not stimulate the same level of cytosolic antioxidant response as in earlier treatment. This was expected based on our previous findings [21].

The expression pattern of genes for GST demonstrated a clear involvement of detoxification-related pathways in the response to *N. ceranae* infection (Figure 4C). The infected control group showed increased GST gene expression, indicating that infection induced detoxification and antioxidant mechanisms associated with the neutralization of oxidative damage products. This is in agreement with the known role of glutathione S-transferase in cellular detoxification and protection against oxidative stress [54,55,58]. Grape pomace supplementation generally reduced GST gene expression in infected bees, particularly in groups treated at the time of infection or after infection. This suggests that grape pomace extract may reduce the need for enhanced GST-mediated detoxification, possibly by lowering the intensity of oxidative and metabolic stress associated with *N. ceranae* infection, similarly to other infections in general [23]. The GP1N group showed a broader distribution of GST expression values, indicating a heterogeneous response among bees receiving preventive supplementation, considering that GST expression is known to be influenced by diet [59]. This variability may reflect individual differences in infection dynamics and metabolism of bioactive compounds, which is not unexpected in honey bees [2,60].

The expression levels of genes for MnSOD (Figure 4D), encoding mitochondrial manganese superoxide dismutase, showed one of the clearest infection-associated responses. The infected control group exhibited the highest MnSOD expression and substantial variability, indicating that *N. ceranae* infection strongly affected mitochondrial redox homeostasis. This is particularly relevant because *N. ceranae* infection is known to disrupt host energy metabolism [3,61,62,63], and mitochondria represent a major intracellular source of reactive oxygen species under metabolic stress. In grape pomace-supplemented groups, MnSOD expression was generally lower than in the infected control. The GP and GP1N groups showed particularly low expression levels, suggesting that the extract did not impose mitochondrial oxidative stress in non-infected bees and that preventive supplementation may reduce the need for strong mitochondrial antioxidant activation after the infection. The GP3N group showed moderately higher MnSOD expression than GP and GP1N, but still remained clearly below the infected control, indicating a controlled mitochondrial antioxidant response when the extract was administered at the time of infection.

## 4. Conclusions

Grape pomace (GP) extract showed potential to improve honey bee survival and reduce *Nosema ceranae* infection intensity. In this study, a single concentration of 200 µg/g was tested, and for additional information regarding efficacy, safety, and dose dependency of the extract, a comprehensive assessment including multiple dose levels is required. The effects of the extract in this study were more pronounced when applied before or together with *Nosema* inoculation rather than as a post-inoculation treatment. The results suggest that GP acts primarily through indirect mechanisms, possibly involving gut health and microbiota modulation as well as antioxidant mechanisms. A clear immunostimulatory effect was observed in non-infected bees, where GP did not prevent infection-induced immune suppression, showing greater preventive (immunostimulatory) than therapeutic potential. The expression of antioxidant-related genes indicated that *N. ceranae* infection induced a pronounced redox response in honey bees, suggesting activation of antioxidant and detoxification pathways in response to infection-associated oxidative stress. Grape pomace supplementation generally reduced the expression of antioxidant-related genes, indicating that the extract lowered the oxidative burden caused by infection. The low expression of CAT, Cu/ZnSOD, GST, and MnSOD in the non-infected (GP) group suggests that the extract itself did not induce oxidative stress in healthy bees. Our findings suggest that grape pomace extract may act as a redox-modulatory supplement in honey bees, attenuating the oxidative stress associated with *N. ceranae* infection (and possibly other pathogens) and contributing to improved physiological resilience. However, bees were analyzed only on day 15, which presents endpoint-associated effects rather than a temporal dynamics study. Moreover, it is necessary to evaluate its effects in combination with other supplements that have more immunostimulatory and anti-*Nosema* effects in more realistic field (hive) conditions.

## Figures and Tables

**Table 1:** Primers used for qPCR analyses.

Primer	Sequence 5′–3′	Reference
Cu/ZnSOD-F	TCAACTTCAAGGACCACATAGTG	[34]
Cu/ZnSOD-R	ATAACACCACAAGCAAGACGAG	[34]
MnSOD-F	GTCGCCAAAGGTGATGTCAATAC	[34]
MnSOD-R	CGTCTGGTTTACCGCCATTTG	[34]
GST-F	AGGAGAGGTGTGGAGAGATAGTG	[34]
GST-R	CGCAAATGGTCGTGTGGATG	[34]
CAT-F	TTCTACTGTGGGTGGCGAAAG	[34]
CAT-R	GTGTGTTGTTACCGACCAAATCC	[34]
VgMC-F	AGTTCCGACCGACGACGA	[35]
VgMC-R	TTCCCTCCCACGGAGTCC	[35]
Abaecin-F	CAGCATTCGCATACGTACCA	[36]
Abaecin-R	GACCAGGAAACGTTGGAAAC	[36]
ApidNT-F	TTTTGCCTTAGCAATTCTTGTTG	[35]
ApidNT-R	GTAGGTCGAGTAGGCGGATCT	[35]
Defensin-F	TGCGCTGCTAACTGTCTCAG	[36]
Defensin-R	AATGGCACTTAACCGAAACG	[36]
Hymenopt-F	CTCTTCTGTGCCGTTGCATA	[36]
Hymenopt-R	GCGTCTCCTGTCATTCCATT	[36]
actin-F	TTGTATGCCAACACTGTCCTTT	[35]
actin-R	GGCGCGATGATCTTAATTT	[35]

F, forward; R, reverse; GST, glutathione S-transferase; Cu/ZnSOD, cytoplasmic Cu-Zn superoxide dismutase; MnSOD, mitochondrial Mn superoxide dismutase; CAT, catalase; VgMC, vitellogenin; actin, beta-actin.

## Data Availability

The original contributions presented in this study are included in the article. Further inquiries can be directed to the corresponding author.
